# Molecular and Proteomic Analyses of Effects of Cadmium Exposure on the Silk Glands of *Trichonephila clavata*

**DOI:** 10.3390/ijms26020754

**Published:** 2025-01-17

**Authors:** Zhaowentao Song, Zhiyu Song, Wei Liu, Bo Lyu

**Affiliations:** 1 School of Biological Sciences, University of Auckland, Auckland 1010, New Zealand; 2 College of Urban and Environment Sciences, Hunan University of Technology, Zhuzhou 412007, China; 3 Division of Plant Science and Technology, University of Missouri, Columbia, MO 65211, USA

**Keywords:** cadmium, silk glands, spider, proteome

## Abstract

Cadmium (Cd) is a pervasive heavy metal pollutant released into the environment through industrial activities such as mining, smelting, and agricultural runoff. This study aimed to investigate the molecular and metabolic impacts of Cd exposure on the silk glands of *Trichonephila clavata*, a species renowned for producing silk with exceptional mechanical properties. Cd accumulation in spider bodies and silk glands was significantly higher in the low- and high-Cd groups compared to controls, with a dose- and time-dependent increase. Oxidative stress markers, including superoxide dismutase, glutathione peroxidase, peroxidase, and malondialdehyde, were significantly elevated, indicating a robust stress response. Proteomic analysis identified 2498 proteins, with 227 differentially expressed between Cd-treated and control groups. Key metabolic pathways, including glutathione metabolism, cysteine and methionine metabolism, and amino acid biosynthesis, were significantly disrupted. Downregulation of enzymes such as glutathione synthase and S-adenosylmethionine synthetase highlighted oxidative imbalance and impaired sulfur metabolism, indicating disruptions in redox homeostasis and energy metabolism critical for silk production. These findings demonstrate that Cd exposure alters oxidative stress responses, disrupts key metabolic pathways, and impairs silk gland functionality at multiple molecular levels. This study advances the understanding of the impact of heavy metal stress on spider physiology and provides a foundation for further research on the ecological implications of Cd contamination.

## 1. Introduction

Environmental contamination by heavy metals is a growing concern worldwide, posing significant threats to ecosystems and biodiversity [1]. Among these pollutants, cadmium (Cd) is a toxic heavy metal widely released into the environment through industrial activities such as mining, smelting, and agricultural fertilization [2,3,4]. Due to its high mobility in soil and water, Cd can readily enter food chains and accumulate in living organisms, where it poses significant risks to both ecological and human health [5,6]. In biological systems, Cd exposure leads to severe cellular disruptions, including oxidative stress, interference with essential enzymes, and perturbations in nutrient homeostasis [7,8]. It can substitute for essential metal ions in biochemical pathways, further amplifying its toxic effects [5]. Long-term Cd contamination has been associated with reduced biodiversity, altered population dynamics, and impaired ecosystem services [9,10]. At the cellular level, Cd induces DNA damage, disrupts cell cycle regulation, and triggers apoptotic pathways, resulting in significant physiological and developmental impairments [11,12]. Furthermore, its persistence in the environment, combined with its non-degradability, exacerbates its impact over time [3]. Understanding how organisms respond to Cd stress at molecular, cellular, and systemic levels is critical for assessing its ecological consequences.

Spider silk, produced by the silk glands of spiders, is an extraordinary natural biomaterial known for its remarkable mechanical properties, including high tensile strength, elasticity, and toughness [13,14,15]. Spiders from the genus *Trichonephila* (e.g., *T. clavata*) are particularly noteworthy for their orb-weaving abilities and the production of complex silk proteins, which serve a variety of ecological functions [16,17]. Orb-weaving spiders possess at least four types of silk glands, each secreting silk with distinct biochemical compositions and specialized functions, such as gluey threads for capturing prey, fibrous silk for constructing egg sacs, and silk for building overwintering shelters [18,19,20]. Spider silk primarily consists of proteins, predominantly made up of amino acids such as alanine, glycine, and serine [21]. The synthesis of spider silk is a metabolically intensive process, requiring precise coordination of amino acid metabolism, protein synthesis, and energy production [20]. Given the metabolic demands of silk production, it is highly sensitive to environmental stressors, including heavy metal contamination [22,23]. While extensive research has focused on the material properties of spider silk [24,25], there is limited understanding of how environmental chemical stressors such as Cd influence the molecular and cellular processes underlying silk synthesis. Previous studies have shown that certain metals, like zinc, titanium, and aluminum, can infiltrate silk proteins and enhance their tensile strength in vitro [26,27]. However, the effects of Cd remain poorly understood. It is indicated that Cd-induced toxicity may decrease the resources available for web construction, as spiders must divert energy toward detoxification instead of silk production [22]. As bioaccumulators of heavy metals, spiders provide a unique model for studying the ecological and physiological impacts of toxic stressors [28,29]. Yet the mechanisms through which Cd affects silk gland function and silk protein synthesis remain largely unexplored.

Oxidative stress is one of the primary mechanisms by which Cd exerts its toxicity, triggering a cascade of cellular disruptions [7]. Cd exposure generates excessive reactive oxygen species (ROS), leading to an imbalance in redox homeostasis [30,31]. This oxidative stress damages cellular macromolecules, including lipids, proteins, and DNA, and compromises essential biological processes [32]. In spiders, where metabolically active silk glands are critical for silk production, oxidative stress can have profound effects on cellular function and silk synthesis [23]. The silk glands, as energy-intensive tissues, are particularly vulnerable to disruptions in metabolic balance and oxidative damage [32,33]. To combat oxidative stress, organisms rely on pathways such as glutathione metabolism, amino acid biosynthesis, and one-carbon metabolism [5]. For instance, glutathione, a key antioxidant, plays a pivotal role in neutralizing ROS and protecting cells from oxidative damage [34]. Amino acid biosynthesis is essential for providing the building blocks for protein synthesis, including spider silk proteins, while one-carbon metabolism supports methylation reactions and nucleotide biosynthesis critical for maintaining DNA integrity and gene expression [20,22]. In the context of Cd toxicity, these pathways are often dysregulated, further exacerbating oxidative damage and impairing cellular function [5,6,11]. Studies in other organisms have shown that Cd-induced oxidative stress can downregulate key metabolic enzymes and disrupt cellular homeostasis, yet the specific impacts on spider silk glands remain poorly understood.

Given the intricate metabolic and cellular processes involved in spider silk synthesis and the vulnerability of silk glands to oxidative and metabolic stress, we hypothesize that Cd exposure significantly impairs silk gland functionality in *Trichonephila clavata*. Specifically, Cd-induced oxidative stress disrupts key antioxidant pathways, including glutathione metabolism, and suppresses amino acid biosynthesis, leading to reduced availability of essential building blocks for silk protein synthesis. Furthermore, we hypothesize that Cd affects the expression of silk protein-related genes and enzymes critical for protein folding and assembly, resulting in compromised silk production. By integrating molecular, proteomic, and metabolic analyses, we seek to investigate the mechanisms through which Cd disrupts silk gland function and highlight the broader ecological implications of heavy metal contamination on spiders. This research provides a foundation for understanding the physiological stress responses of spiders under Cd exposure and their potential as bioindicators in contaminated environments.

## 2. Results

### 2.1. Cadmium Accumulation in Spiders and Silk Glands

**Figure 1:**
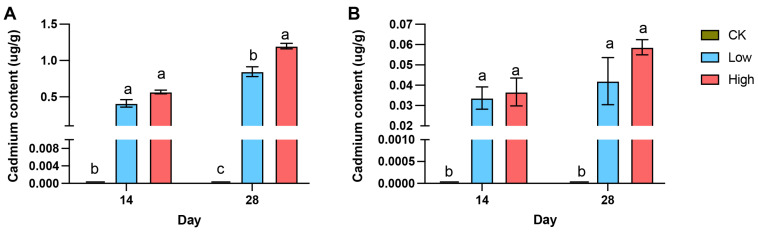
Cadmium (Cd) accumulation in the bodies and silk glands of *T. clavata* under different treatments and time points. (**A**) Cd content in whole-body samples from control (CK), low-Cd, and high-Cd treatment groups at 14 and 28 days. (**B**) Cd content in the silk glands across the same groups and time points. Bars represent the mean ± standard error (SE), and different letters indicate statistically significant differences (*p* < 0.05) among groups and time points.

Cd content in spider bodies and silk glands increased significantly in response to Cd exposure, with notable differences across treatment groups and time points. In whole-body samples (Figure 1A; Table S1), Cd levels in the low- and high-Cd groups were significantly elevated compared to the control (CK) group at both 14 and 28 days (*p* < 0.001). The high-Cd group showed a marked increase over time, with Cd content reaching 1.19 ± 0.05 μg/g on day 28 (*p* < 0.001 compared to CK). Similarly, in the silk glands (Figure 1B; Table S1), Cd accumulation was higher in the low- and high-Cd groups relative to CK, with the high-Cd group showing a progressive increase from 0.037 ± 0.001 μg/g on day 14 to 0.059 ± 0.005 μg/g on day 28 (*p* < 0.01).

### 2.2. Concentrations of Oxidative Stress Markers

**Figure 2:**
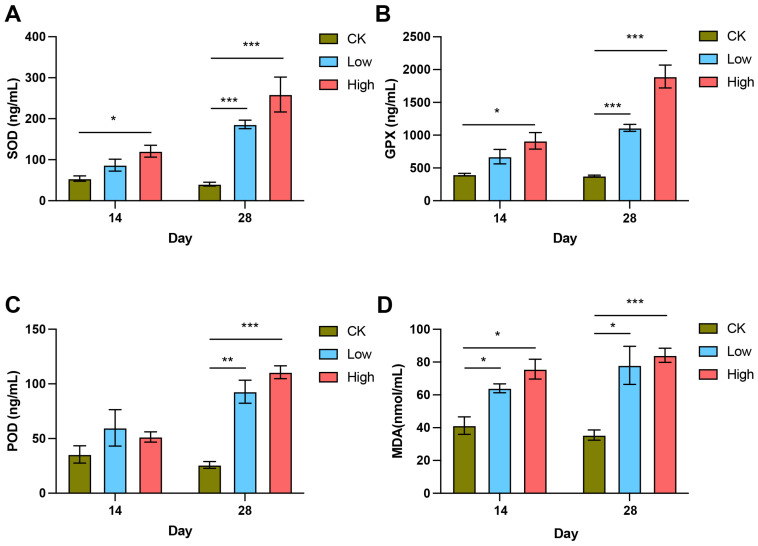
Concentrations of oxidative stress markers in *T. clavata* under Cd exposure. (**A**) Superoxide dismutase (SOD) concentration, (**B**) glutathione peroxidase (GPX) concentration, (**C**) peroxidase (POD) concentration, and (**D**) malondialdehyde (MDA) concentration in the control (CK), low-Cd, and high-Cd groups after 14 and 28 days of exposure. Bars represent the mean ± standard error (SE), and asterisks (*) indicate statistically significant differences (* *p* < 0.05, ** *p* < 0.01, *** *p* < 0.001) between groups and time points.

The concentrations of oxidative stress-related markers, including SOD, POD, GPX, and MDA, showed significant increases in response to Cd exposure across the treatment groups and time points (Figure 2). SOD concentration (Figure 2A) increased significantly in both the low- and high-Cd groups compared to the CK group, with the high-Cd group showing a particularly notable elevation at 28 days (*p* < 0.001). GPX concentrations (Figure 2B) also increased markedly, with the high-Cd group exhibiting the highest levels at both 14 and 28 days, nearly doubling compared to the CK group (*p* < 0.001). Similarly, POD concentrations (Figure 2C) were higher in the Cd-treated groups, with the high-Cd group displaying a significant increase by 28 days (*p* < 0.001). Consistent with these changes, MDA concentrations (Figure 2D), an indicator of lipid peroxidation and oxidative damage, were significantly elevated in the Cd-treated groups. The high-Cd group showed the most pronounced increase (*p* < 0.001).

### 2.3. Proteomic Analysis of Spider Silk Glands

**Figure 3:**
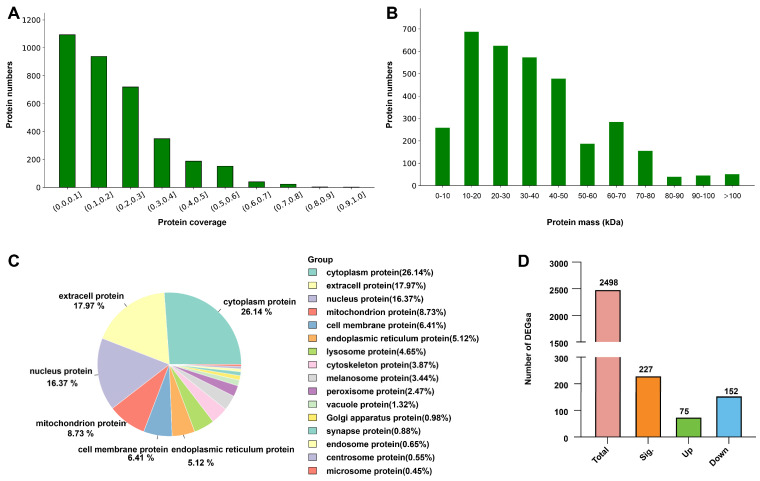
Proteomic analysis of silk glands in *T. clavata* under Cd exposure. (**A**) Protein coverage distribution of identified proteins. (**B**) Molecular weight distribution of identified proteins. (**C**) Subcellular localization of identified proteins. (**D**) Differentially expressed proteins between the high-Cd and control (CK) groups.

Label-free proteomic analysis generated a total of 198,371 spectra, with 65,271 matching spectra, resulting in the identification of 18,192 peptides and 2498 proteins. The majority of peptides consisted of 8–12 amino acid residues, consistent with high-quality sequencing data. Protein coverage analysis revealed that most identified proteins exhibited moderate coverage levels, with a distribution across functional categories and molecular pathways (Figure 3A). The molecular weight distribution of identified proteins ranged broadly, with most clustering between 10 and 50 kDa, typical for structural proteins and enzymes (Figure 3B). Subcellular localization analysis showed that the identified proteins were predominantly cytoplasmic (26.14%), extracellular (17.97%), and nuclear (16.37%), followed by mitochondrial proteins (8.73%), with smaller proportions from the cell membrane (6.41%), endoplasmic reticulum (5.12%), and lysosome (4.65%) (Figure 3C). Differential expression analysis revealed 227 DEPs (e.g., ribosomal proteins, glutathione synthase, and glycine hydroxymethyltransferase), including 75 significantly upregulated and 152 significantly downregulated proteins, in the high-Cd group compared to the CK group (Figure 3D).

### 2.4. GO and KEGG Functional Annotation of Differentially Expressed Proteins

**Figure 4:**
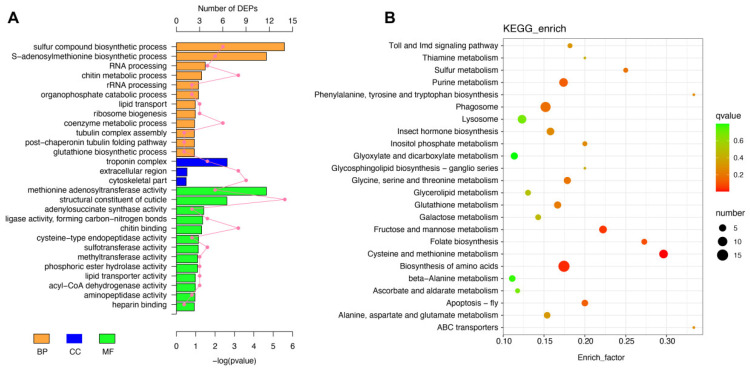
GO and KEGG pathway enrichment analyses of DEPs in the silk glands of *T. clavata* under Cd exposure. (**A**) GO enrichment analysis of DEPs categorized into biological processes (BPs), cellular components (CCs), and molecular functions (MFs). The polyline represents the number of DEPs, while the bar plot illustrates their statistical significance (*p*-value). (**B**) KEGG pathway analysis of significant enriched signaling pathways.

Functional annotation of the DEPs revealed significant enrichment in biological processes, cellular components, molecular functions, and metabolic pathways, highlighting the impact of Cd exposure on silk gland function (Figure 4). GO analysis showed that DEPs were predominantly associated with biological processes (BPs) such as the glutathione biosynthetic process, sulfur compound biosynthetic process, and RNA processing. Key cellular components (CCs) included the cytoskeletal part and ribosomal complex, while enriched molecular functions (MFs) included glutathione transferase activity, lipid transporter activity, and cysteine-type endopeptidase activity, reflecting the impact of Cd-induced stress on protein synthesis, metabolic regulation, and structural maintenance (Figure 4A). KEGG pathway enrichment analysis identified several significantly affected pathways, including glutathione metabolism, cysteine and methionine metabolism, amino acid biosynthesis, sulfur metabolism, and Toll and Imd signaling pathways (Figure 4B). Notably, pathways such as glutathione metabolism and cysteine and methionine metabolism underscore the role of antioxidant defense and sulfur compound biosynthesis in mitigating Cd-induced oxidative stress.

### 2.5. Metabolic and Stress Response Disruptions in Spider Silk Glands

**Figure 5:**
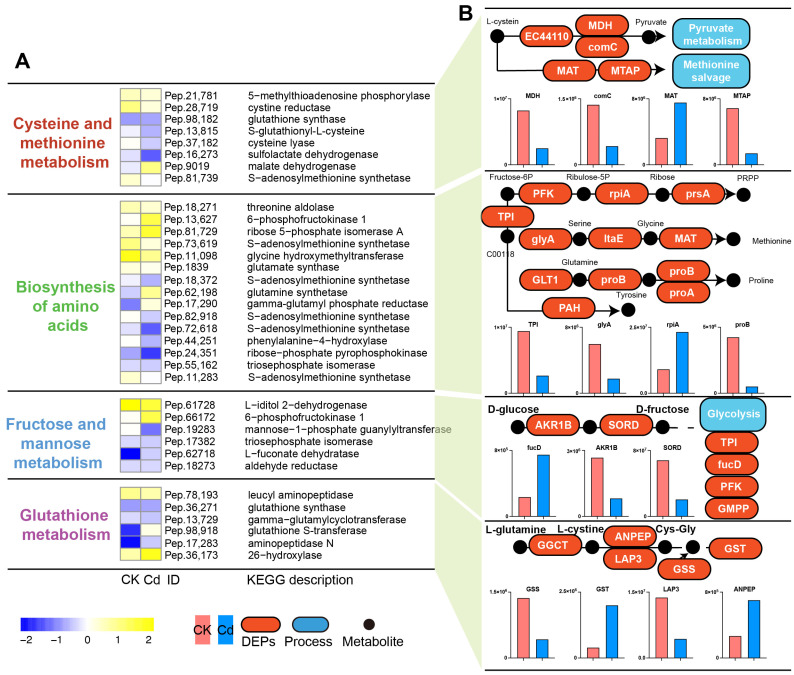
Key KEGG pathway annotations of DEPs in the silk glands of *T. clavata* under Cd exposure. (**A**) Heatmap showing the relative abundance of DEPs involved in key metabolic pathways, including cysteine and methionine metabolism, biosynthesis of amino acids, fructose and mannose metabolism, and glutathione metabolism, across experimental groups. (**B**) Representative KEGG pathway modules mapped from DEPs. Bar plots show the relative expression levels of key proteins involved in these pathways across control (CK) and Cd-treated groups.

KEGG pathway analysis of the DEPs revealed significant enrichment in pathways related to metabolism and stress response in the silk glands of *T. clavata* under Cd exposure. Heatmap analysis (Figure 5A) highlighted changes in the relative abundance of key proteins involved in cysteine and methionine metabolism, the biosynthesis of amino acids, fructose and mannose metabolism, and glutathione metabolism. These pathways are critical for maintaining redox homeostasis, amino acid biosynthesis, and cellular energy metabolism. Representative KEGG pathway modules mapped from these DEPs (Figure 5B) showed disruptions in several key processes. For instance, glutathione synthase (GSS), S-adenosylmethionine synthetase (MAT), and 5-methylthioadenosine phosphorylase (MTAP) were significantly downregulated in the high-Cd group, indicating suppression of glutathione metabolism and methionine salvage pathways. Additionally, proteins such as glycine hydroxymethyltransferase (glyA), gamma-glutamyl phosphate reductase (proA), and glutamate synthase (GLT1) involved in amino acid biosynthesis were also downregulated, suggesting that Cd stress inhibits amino acid availability, necessary for silk protein synthesis. Enzymes related to fructose and mannose metabolism, such as 6-phosphofructokinase (PFK) and ribose-5-phosphate isomerase (rpiA), showed decreased abundance, potentially disrupting energy metabolism.

### 2.6. Gene Expression Analysis by qPCR

**Figure 6:**
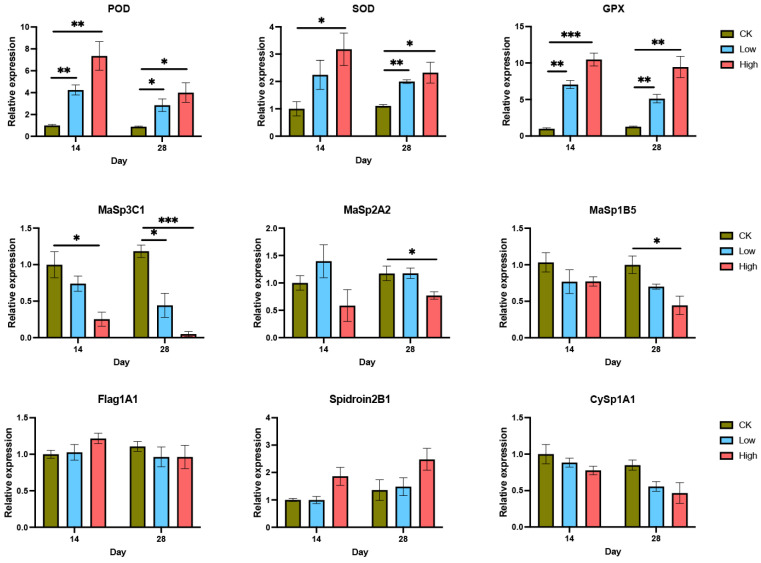
Gene expression analysis of antioxidant and silk protein-related genes in the silk glands of *T. clavata* under Cd exposure. Bars represent the mean ± standard error (SE), and asterisks indicate statistically significant differences among groups and time points (* *p* < 0.05, ** *p* < 0.01, *** *p* < 0.001).

qPCR analysis revealed significant changes in the expression of key antioxidant and silk protein-related genes in the spider silk glands across the three treatment groups (CK, low Cd, and high Cd) and two time points (14 and 28 days) (Figure 6). Among antioxidant genes, the relative expression of POD, SOD, and GPX was markedly elevated in response to Cd exposure. For POD, expression increased significantly in both low- and high-Cd groups compared to CK at both 14 and 28 days, with the high-Cd group showing the most substantial upregulation (*p* < 0.05). Similarly, SOD expression was upregulated in both Cd-treated groups, with a peak in the high-Cd group on day 28 (*p* < 0.05). GPX expression followed a similar trend, showing a dose- and time-dependent increase, particularly in the high-Cd group (*p* < 0.01). For silk protein-related genes, *MaSp1B5*, *MaSp2A2*, and *MaSp3C1* exhibited downregulation in response to Cd exposure, with the high-Cd group showing significant reductions at both time points (*p* < 0.05). Other silk protein genes, including *Flag1A1*, *Spidroin2B1*, and *CySp1A1*, were not altered following 14 d and 28 d treatments.

## 3. Discussion

This study showed that Cd exposure induces significant oxidative stress in the silk glands of *T. clavata* while simultaneously suppressing the expression of key genes and proteins involved in silk protein synthesis and amino acid metabolism. Compared with wandering and wolf spiders, web-weaving spiders are significant accumulators of metals, primarily ingesting them through prey, and must allocate energy toward detoxification rather than other physiological processes like web construction [35]. Excess metals are often neutralized in the midgut through mineral concretions or by binding to metallothioneins [36]. As a result, the midgut typically shows the highest cadmium concentration, while other organs like venom glands, reproductive tissues, and ganglia have relatively lower cadmium levels but may be more sensitive to metal-induced stress [22,37]. Cd exposure significantly induced oxidative stress in *T. clavata*, as evidenced by elevated expression levels of antioxidant genes and enzymatic contents such as SOD and GPX. This finding aligns with prior studies on various arthropods, which reported that heavy metals stimulate the generation of ROS, including superoxide radicals (O_2_^−^), hydrogen peroxide (H_2_O_2_), and nitric oxide (NO), leading to oxidative stress [38]. High ROS levels are known to damage cellular structures, disrupt metabolic functions, and trigger apoptosis or necrosis [39,40]. In response, organisms activate antioxidant systems to neutralize ROS and maintain homeostasis. SOD, for example, serves as a natural ROS scavenger, converting superoxide radicals into oxygen and hydrogen peroxide to minimize oxidative damage [41]. Our findings are consistent with studies on the pond wolf spider (*Pirata subpiraticus*), where Cd stress significantly increased GST and SOD activity, suggesting an immediate protective response against acute Cd toxicity [42]. However, prolonged Cd exposure can overwhelm the antioxidant system, leading to sustained ROS accumulation and further cellular damage. This is evident in our proteomic data, which revealed downregulation of key enzymes in the glutathione metabolism pathway, such as GSS, under 28 d Cd stress. Such disruptions indicate resource depletion for sustaining antioxidant defenses, similar to findings in other spider species like *Xerolycosa nemoralis*, where long-term exposure increased glutathione-dependent enzyme activity but eventually led to oxidative imbalance [43]. Altered POD and SOD expression levels in the ovaries of spiders under prolonged Cd exposure have been reported, indicating reproductive toxicity [44]. Similarly, we observed significant changes in the antioxidant system of silk glands, a metabolically active tissue essential for silk protein synthesis. This suggests that Cd stress may have broader implications for spider physiology, potentially impairing both survival and reproduction.

Spiders are well known for their ability to produce silk, a feature that defines their ecological adaptability. All spiders have specialized silk glands used for producing egg sacs, draglines, and traps for capturing prey [20]. Spider silk proteins are characterized by a unique amino acid composition, with small side-chain amino acids comprising over 50% of their structure. Tubular glands produce silk for egg sacs, while major ampullate glands are responsible for the production of frame threads essential for web-building [45]. This metabolically intensive process requires a significant energy investment, particularly in amino acid synthesis. Under Cd stress, however, spiders may allocate less energy toward silk production, as seen in the observed downregulation of silk protein-related genes and metabolic pathways [22]. In our study, KEGG analysis revealed that key enzymes such as GSS, MTAP, and glycine GNMT were significantly downregulated under Cd exposure. These enzymes are critical for the regulation of cysteine, methionine, glycine, serine, and threonine metabolism. Similar findings have been reported in other spiders, where Cd stress suppresses amino acid metabolism, further confirming the inhibitory effects of heavy metals on silk protein synthesis [46]. Specifically, proteins or enzymes such as glutathione synthase, S-adenosylmethionine synthetase, and 5-methylthioadenosine phosphorylase, which regulate glutathione metabolism and methionine salvage pathways, were significantly downregulated in the high-Cd group. This suppression impairs redox homeostasis, exacerbating oxidative stress and metabolic disruption [28,43]. Similarly, the downregulation of glycine hydroxymethyltransferase, gamma-glutamyl phosphate reductase, and glutamate synthase significantly impacts amino acid metabolism, which is crucial for silk protein synthesis. Glycine hydroxymethyltransferase is vital for producing glycine and serine, key components of silk proteins, while gamma-glutamyl phosphate reductase is involved in proline biosynthesis, contributing to silk elasticity and strength [20]. Interestingly, some studies have observed species-specific differences in response to heavy metal stress. For instance, amino acid levels such as alanine, histidine, and phenylalanine in the silk glands of *Steatoda grossa* remained unaffected under Cu^2+^ and Cd^2+^ exposure [22]. In contrast, our findings suggest that *T. clavata* reallocates metabolic resources under prolonged Cd stress, prioritizing detoxification and stress responses over silk production. This shift in resource allocation aligns with the energy-intensive nature of amino acid synthesis and the metabolic burden imposed by oxidative stress. Future studies should explore these interspecies differences under varying experimental conditions to better understand the broader implications of heavy metal stress on spider silk production and ecological fitness.

Our GO enrichment revealed that DEPs were predominantly associated with biological processes such as sulfur compound biosynthesis, glutathione metabolism, and amino acid synthesis, as well as cellular components like cytoskeletal structures and ribosomal complexes. These findings indicate that Cd stress impacts fundamental processes critical for maintaining silk gland functionality, including protein synthesis, structural stability, and metabolic balance [5,7,11]. KEGG analysis further supported this by identifying significant disruptions in pathways such as glutathione metabolism, cysteine and methionine metabolism, and amino acid biosynthesis. For instance, GSS and MAT, two enzymes crucial for maintaining redox homeostasis and methionine cycling [28,47], were downregulated in response to Cd exposure. These changes impair the gland’s ability to neutralize ROS, leading to oxidative stress and cellular damage. At the cellular level, the disruption of cytoskeletal and ribosomal components may further compromise silk gland functionality [48]. Disruptions in cytoskeletal organization and ribosomal biogenesis emerged as significant findings unrelated to oxidative stress or metabolic pathways. Cytoskeletal proteins, which were enriched in GO terms for structural integrity, are critical for maintaining cellular architecture and intracellular transport. Under Cd stress, the dysregulation of these proteins could impair vesicle trafficking essential for silk protein storage and secretion. Similarly, the downregulation of ribosomal proteins and pathways associated with ribosome biogenesis suggests that Cd exposure hinders translation efficiency [49], reducing the overall capacity for silk protein production. The combined effects of metabolic disruption, oxidative stress, and structural impairment create a feedback loop that exacerbates cellular dysfunction in the silk glands. This is consistent with the enrichment of signaling pathways like the Toll and Imd pathways observed in the KEGG analysis, which are activated in response to cellular stress and infection but may become maladaptive under prolonged Cd exposure. Persistent activation of these pathways could lead to energy depletion and further diversion of resources away from silk protein synthesis [22,50].

The downregulation of silk protein-related genes, such as *MaSp1B5* and *MaSp2A2*, likely has significant consequences for both the quality and quantity of silk produced. Reduced gene expression directly limits the synthesis of major ampullate spidroins, which are critical for the tensile strength and elasticity of silk [51]. This may result in weaker silk with compromised mechanical properties, affecting its ecological functions such as web stability and prey capture efficiency. Additionally, decreased production of silk proteins could reduce the overall quantity of silk, potentially impacting behaviors like web-building, egg sac construction, and other survival strategies [20,52]. Overall, this study reveals that Cd exposure induces oxidative stress and disrupts key metabolic pathways, including amino acid and sulfur metabolism, ultimately suppressing silk protein-related gene expression and impairing silk gland functionality in *T. clavata*. These findings provide foundational insights into the molecular mechanisms underlying heavy metal stress in spiders and its broader ecological implications. However, future research should integrate mechanical testing, behavioral studies, and advanced imaging techniques to comprehensively assess the effects of Cd on spider silk performance and ecological adaptability.

## 4. Materials and Methods

### 4.1. Spider Preparation

Mature female *T. clavata* spiders with comparable body sizes (12.8 ± 0.5 mm) and weights (0.65 ± 0.1 g) were collected from farmland and grass shrub areas free from heavy metal pollution in Dali, Yunnan Province, China. The spiders were housed individually in plastic boxes (12 cm in diameter × 10 cm in height) equipped with wooden frames to support web spinning and a moistened cotton ball to maintain humidity. They were reared in an artificial climate chamber under controlled conditions of 25 °C, 75% relative humidity, and a 10:14 light/dark photoperiod. Based on the Cd concentration in the *Drosophila melanogaster* feeding medium, the spiders were divided into three groups: a control group (CK), where flies were reared on Cd-free medium; a low-Cd-concentration group (low), where flies were reared on medium containing 0.3 mg/L Cd; and a high-Cd-concentration group (high), where flies were reared on medium containing 1.5 mg/L Cd (GB2762-2017) [53]. Each spider was provided with 10 flies daily, along with water ad libitum via a moistened cotton ball, for treatment durations of two and four weeks. Spiders were anesthetized using CO_2_ and dissected on ice, with silk glands (i.e., major ampullate glands) carefully isolated under a dissecting microscope. The dissected glands were immediately placed in liquid nitrogen and preserved at −80 °C for RNA, protein, and molecular analyses.

### 4.2. Cadmium Determination

To determine Cd concentrations, spider and gland samples were subjected to a standardized digestion process. Prior to digestion, digestion tubes were soaked overnight in nitric acid (HNO_3_), thoroughly rinsed with ddH_2_O, and dried at 110 °C. For each sample, 3 spiders and 10 ampullate glands (in triplicate) were pooled and placed into digestion tubes. Each sample was treated with 1.5 mL of concentrated HNO_3_ and 4.5 mL of concentrated HCl and digested using a digestion instrument under a two-step heating protocol (90 °C 1 h and 120 °C 2 h). After digestion, the resulting clear solution was diluted to 50 mL with ddH_2_O and analyzed for Cd content using inductively coupled plasma spectrometry (Shimadzu ICPE, Shimadzu Corporation, Kyoto, Japan) at a wavelength of 228.3 nm. Quality control was ensured by including reagent blanks and certified reference materials in each batch of samples, with recovery rates for Cd ranging between 95% and 105%.

### 4.3. Enzymatic Concentration Determination

The enzymatic concentrations were measured in whole spiders from the three experimental groups. Each sample was accurately weighed and processed in homogenizers with the addition of 2 mL of PBS (pH 7.2), followed by homogenization on ice for 3–5 min. The homogenates were then centrifuged at 10,000× *g* for 10 min at 4 °C, and the supernatants were collected, aliquoted, and stored at −80 °C until further analysis. The levels of superoxide dismutase (SOD), glutathione peroxidase (GPX), peroxidase (POD), and malondialdehyde (MDA) were determined using enzyme-linked immunosorbent assay (ELISA) kits from Nanjing Jiancheng Bioengineering Institute (Nanjing, China) following the manufacturer’s instructions. Absorbance was measured at 450 nm using a BioTek ELx808 microplate reader (Agilent Technologies, Santa Clara, CA, USA). To ensure accuracy and reproducibility, each sample was analyzed in triplicate for all assays.

### 4.4. Proteomic Analysis

Proteomic analysis of the major ampullate glands from *T. clavata* spiders in the control (CK) and high-Cd (high) groups was performed as follows. Tissue samples stored at −80 °C were thawed and homogenized in PASP protein lysis buffer containing 100 mM ammonium bicarbonate and 8 M urea (pH 8). The homogenates were centrifuged at 12,000× *g* for 15 min at 4 °C, and the supernatants were collected. The protein samples were reduced with 10 mM dithiothreitol (DTT), precipitated with pre-chilled acetone, and resuspended in a protein dissolution buffer (8 M urea, 100 mM triethylammonium bicarbonate, TEAB, pH 8.5) to obtain solubilized protein. Protein concentrations were determined using a Bradford Protein Assay Kit (Bio-Rad Laboratories, Hercules, CA, USA), with absorbance measured against a standard protein curve. Peptide separation was conducted using a high-resolution liquid chromatography system coupled with a mass spectrometer set at a first-level resolution of 60,000 (200 *m/z*) and a second-level resolution of 15,000 (200 *m/z*). The raw spectral data were analyzed using Proteome Discoverer 2.4 (PD2.4, Thermo Fisher Scientific, Waltham, MA, USA), with protein identification performed against the spider protein database. Differentially expressed proteins (DEPs) were filtered using thresholds of *p* < 0.05 and |log_2_FC| > 1. Gene Ontology (GO) and InterProScan (IPR) functional annotations were performed for all identified proteins, and Kyoto Encyclopedia of Genes and Genomes (KEGG) pathway enrichment analysis was conducted to understand the biological significance of DEPs.

### 4.5. RT-qPCR Analysis

Total RNA was isolated using the TRIzol reagent (Invitrogen, Waltham, MA, USA) according to the manufacturer’s instructions. RNA concentration and purity were measured using a NanoDrop spectrophotometer (Thermo Fisher Scientific, Waltham, MA, USA), and its integrity was confirmed by agarose gel electrophoresis. For cDNA synthesis, 1 µg of total RNA was reverse-transcribed with a PrimeScript RT reagent kit (Takara Bio Inc., Kusatsu, Shiga, Japan) using random primers and oligo-dT. qPCR was carried out on a QuantStudio 5 Real-Time PCR System (Thermo Fisher Scientific, Waltham, MA, USA) using SYBR Green PCR Master Mix (Applied Biosystems, Waltham, MA, USA). Each 20 µL reaction contained 10 µL of SYBR Green mix, 0.4 µL each of forward and reverse primers (10 µM), 2 µL of diluted cDNA, and 7.2 µL of nuclease-free water. Thermal cycling conditions included an initial denaturation at 95 °C for 3 min, followed by 40 cycles of denaturation at 95 °C for 10 s, annealing at 60 °C for 30 s, and extension at 72 °C for 20 s. Gene-specific primers were designed using Primer3 software (Table S2). The relative expression levels of target genes, including silk protein genes (e.g., *MaSp3C1* and *Flag1A1*) and antioxidant genes (e.g., *SOD* and *GPX*), were calculated using the 2^−ΔΔCt^ method [54], with GAPDH used as the internal reference gene. All reactions were performed in triplicate, and the mean values were used for statistical analysis.

### 4.6. Data Analysis

Statistical analysis was performed using GraphPad Prism (v9.0) software. Normality and homogeneity of variances were tested using the Shapiro–Wilk and Levene tests, respectively. For comparisons between two groups, a two-tailed Student’s *t*-test was applied. For multiple-group comparisons, one-way analysis of variance (ANOVA) followed by Tukey’s post hoc test was used to determine significant differences. For non-parametric data, the Kruskal–Wallis test followed by Dunn’s post hoc test was applied. The significance threshold was set at *p* < 0.05. For functional enrichment analysis, GO and KEGG annotations were statistically assessed using Fisher’s exact test or hypergeometric tests, with adjusted *p*-values calculated using the Benjamani–Hochberg method.

## 5. Conclusions

This study provides novel insights into the molecular and metabolic disruptions caused by Cd exposure in the silk glands of *T. clavata*. By integrating molecular and proteomic analyses, we demonstrated that Cd exposure induces significant oxidative stress, disrupts key metabolic pathways such as amino acid and sulfur metabolism, and suppresses the expression of silk protein-related genes like *MaSp1B5* and *MaSp2A2*. Our findings underscore the complex interplay between oxidative stress, metabolic alterations, and silk protein biosynthesis, offering a foundational understanding of how heavy metal exposure affects spider silk gland performance. This research highlights the potential of spider silk glands as bioindicators for environmental contamination and contributes to the broader field of ecotoxicology.

## Data Availability

All data generated or analyzed during this study are included in the article and its Supplementary Materials. For additional information, please contact the corresponding authors.

## Supplementary Materials

The following supporting information can be downloaded at https://www.mdpi.com/article/10.3390/ijms26020754/s1.
