# Supplementary material for: Molecular and Proteomic Analyses of Effects of Cadmium Exposure on the Silk Glands of Trichonephila clavata
Source: Int J Mol Sci. 2025 Jan 17;26(2):754. doi: 10.3390/ijms26020754 (PMC11765807; doi:10.3390/ijms26020754)
Supplement: Supplementary file 1 [file ijms-26-00754-s001.zip › ijms-3388624-supplementary.docx]

**Table S1. The concentration of cadmium in spiders**

|  | Whole body (ug/g) | | | Silk gland (ug/g) | | |
| --- | --- | --- | --- | --- | --- | --- |
| Group | CK | Low | High | CK | Low | High |
| 14 | 0e | 0.409±0.051 | 0.567±0.025 | 0e | 0.034±0.005 | 0.037±0.007 |
| 28 | 0e | 0.847±0.067 | 1.198±0.037 | 0e | 0.042±0.012 | 0.059±0.004 |

**Table S2. Primers used for qRT-PCR analysis**

| Gene | Description | Primer |
| --- | --- | --- |
| MaSp2A2 | major ampullate spidroin 2A variant 2 | F: TTGGCGCAAGTAATCCTGGT |
|  |  | R: TGGCTGACAACTTGAGCGAA |
| Flag1A1 | flagelliform spidroin 1A variant 1 | F: TGCTTCTCGTCTTTCGTCCC |
|  |  | R: TCCAATGCTTGCAGAACCCA |
| MaSp1B5 | major ampullate spidroin 1B variant 5 | F: TGCTTCTCGTCTTTCGTCCC |
|  |  | R: TCCAATGCTTGCAGAACCCA |
| MaSp3C1 | major ampullate spidroin 3C variant 1 | F: TGGAGCAGGTGGATTAGGGT |
|  |  | R: GAAGATCCAACACCGCCGTA |
| spidroin2B1 | spidroin 2B variant 1 | F: GCGCAATTGGTGTTGGCATA |
|  |  | R: AGCTTCCGCTCCAACATTCA |
| GPX4 | phospholipid hydroperoxide glutathione peroxidase | F: CGTAGGTTACTGGCTTGGGG |
|  |  | R: GGCTCCTGACCTCCAAACTG |
| CySp1A1 | cylindrical spidroin 1A variant 1 | F: GCGTCTGCATTGTCGACTTC |
|  |  | R: GCTGGGGACGCTAGAAACAT |
| POD | peroxidase | F: CCACATTGGGACGACGAGAA |
|  |  | R: GCGTCCAATTCAGGGTCGTA |
| SOD1 | superoxide dismutase | F: ACGAAACTCCAACTCCCGTC |
|  |  | R: TACCCAAGTCTCCAACGTGC |
| GAPDH | glyceraldehyde 3-phosphate dehydrogenase | F: GATTTGGCCGTATTGGACGC |
|  |  | R: AGTTGGAACACGGAAAGCCA |
